# Evolution and circulation of *Yersinia pestis* in the Northern Caspian and Northern Aral Sea regions in the 20^th^-21^st^ centuries

**DOI:** 10.1371/journal.pone.0244615

**Published:** 2021-02-11

**Authors:** Galina A. Eroshenko, Nikolay V. Popov, Zhanna V. Al’khova, Lyubov M. Kukleva, Alina N. Balykova, Nadezhda S. Chervyakova, Ekaterina A. Naryshkina, Vladimir V. Kutyrev

**Affiliations:** Russian Research Anti-Plague Institute “Microbe”, Federal Service for Surveillance in the Sphere of Consumers Rights Protection and Human Welfare, Saratov, Russian Federation; Zhejiang University, CHINA

## Abstract

According to the whole genome SNP analysis of 38 *Yersinia pestis* strains isolated in the foci of the Northern Caspian and Northern Aral Sea regions in the 20th–early 21st centuries, between 1912 and 2015, the spatial and temporal structure of the 2.MED population of a medieval biovar in this region was determined. A phylogenetic branch 2.MED4 was identified which preceded the 2.MED1 branch that diverged later. 2.MED1 strains became the etiological agent of high-mortality plague outbreaks that occurred in the Northern Caspian region at the beginning of the 20th century. Later in the 20th century, the 2.MED1 branch became widespread in the Caspian Sea region, Caucasus, and vast areas of Central Asia. Based on the data of phylogenetic analysis, as well as epidemiological and epizootiological data, we reconstructed the paths of spread of the 2.MED1 branch in the Northern Caspian Sea region and in the Northern subzone of the Central Asian deserts. It is shown, that the reason for the activation of plague foci in the Northern Caspian region in the second half of the 20th century after a long inter-epizootic period caused by cyclical climate warming was the return of 2.MED1 from the foci of the Northern Aral Sea region. This led to the formation of stable plague foci in the Northern Caspian Sea region and Pre-Caucasus, which manifested epizootic activity in the second half of the 20th and early 21st centuries.

## Introduction

There is a group of natural plague foci located in Russia and Kazakhstan in steppe, semi-desert, and desert zones in the Northern Caspian Sea region. The area of the plague foci at the northern border of the desert subzone stretches eastward, reaching the Northern Aral Sea region, and then descends to the Balkhash region (S1 Fig). In the west, the foci of the North-Western Caspian Sea region meet the foci of Pre-Caucasus [1].

All these foci differ significantly in their species spectrum of host and vectors. The main carrier of plague in the steppe foci is the small souslik *Spermophilus pygmaeus*, the main flea vectors are *Neopsylla setosa* and *Citellophilus tesquorum*. In the foci of the sandy type in the Northern and North-Western Caspian Sea regions, the main carriers are gerbils–midday *Meriones meridianus* and crested *M*. *tamariscinus*, and the flea vectors are *Nosopsyllus laeviceps* and *Xenopsylla conformis*. In the foci of the Northern subzone of deserts in Central Asia, the main carrier is the great gerbil *Rhombomis opimus*, and the vectors are fleas of the genus *Xenopsylla*. Climate aridity increases with the movement from steppe towards semi-desert and desert landscapes. The plague foci of the Caspian and Aral Sea regions, as well as the desert foci of Central Asia, are characterized by clearly defined periods of high activity and rest. During rest periods, there are no epizootics in the foci and no cultures of the plague pathogen *Yersinia pestis* are isolated. The reasons of activation of steppe and desert plague foci after long inter-epizootic periods are mainly associated with climate changes.

In the late 19th and early 20th centuries, there were outbreaks of plague with high mortality rates in the Caspian Sea region [2]. The largest number of cases was registered in the Caspian North-Western steppe, Volga-Ural steppe, and Volga-Ural sandy foci. The incidence continued until the late thirties and early forties, then there was a significant decrease in epidemic and epizootic activity with the onset of a long inter-epizootic period in the mid-20th century. The last epizootic manifestations in the steppe, semi-desert and desert landscapes of the Caspian Sea area in Russia and Kazakhstan before the onset of the inter-epizootic period were registered in the 1940-1950s, including the Ural-Wil steppe focus in 1941, the Volga-Ural sandy focus in 1952, the Volga-Ural steppe focus in 1950, the Caspian North-Western steppe focus in 1954, and the Dagestan plain-piedmont focus in 1956. The end of epizootics was associated with the measures deployed to exterminate rodents, carried out in the territories of those foci, which were unprecedented in scale in the world practice. At the beginning of the second half of the 20th century, in 1963–1979, plague epizootics began to reoccur in the territory of the Caspian lowland and in Pre-Caucasus, including the Volga-Ural sandy focus in 1963, the Dagestan plain-piedmont focus in 1975, the Volga-Ural steppe (Ural-Kushum Interfluve) and Ural-Wil steppe foci in 1978, the Caspian North-Western steppe focus (Caspian lowland) in 1979. The inter-epizootic period ranged from 11 to 37 years. The reasons for the activation of these natural foci of plague in the second half of the 20th century remain unclear.

In another region of the Northern subzone of the Eurasian deserts–in the Northern Aral Sea region in Kazakhstan, plague epizootics were first detected in 1945, after an outbreak of plague with a high mortality rate occurred in the North-Aral desert focus. There is no earlier historical data about the plague in this region. Later, in the second half of the 20th and early 21st century, epidemic and epizootic activity was repeatedly registered in the North-Aral and Aral-Karakum desert foci.

During the period from the beginning of the 20th century till present days, while performing epidemiological and epizootiological monitoring in the natural foci of plague in the Caspian Sea and Aral Sea regions, specialists of the Plague Control System of the Soviet Union, and then Russia have gathered a unique collection of *Y*. *pestis* strains, which chronologically reflects the history of the foci. According to microbiological and molecular genetic studies of a large number of strains, it was revealed that in the foci of the Caspian and Aral Sea regions *Y*. *pestis* of the medieval biovar circulates [1, 3]. The strains of this biovar are highly virulent and epidemiologically significant. In accordance with the genetic nomenclature of the lineages of *Y*. *pestis* evolution, the medieval biovar is designated as 2.MED. Within this lineage, populations 2.MED0, 2.MED1, 2.MED2, 2.MED3 are distinguished [4–7]. The earliest divergent branch– 2. MED0 was detected in the Central-Caucasian high-mountain focus in Russia. It is followed by 2.MED2 and 2.MED3 branches from the foci of China. The youngest branch of the medieval biovar is 2.MED1, which is widely distributed in the Caspian Sea region, Caucasus, and Central Asia in countries such as Russia, Kazakhstan, Uzbekistan, Turkmenistan, Kyrgyzstan, Azerbaijan, Georgia. It is also found in East Asian countries such as China and Mongolia [6, 8]. We assume that the period of dissemination of 2.MED1 is the mid-19^th^-mid-20^th^ centuries. Due to the relatively recent events that occurred here, the availability of detailed epizootiological and epidemiological data, as well as a large collection of *Y*. *pestis* strains, the plague foci of the Northern Caspian and Northern Aral Sea regions are a good model for identifying patterns of spread of medieval biovar in the landscapes of steppes, semi-deserts and deserts of Eastern Europe and Central Asia in the 20th-21st centuries. The objective of this study was: phylogenetic analysis of *Y*. *pestis* strains isolated in different periods of epidemic and epizootic activity in the foci of the Northern Caspian and Northern Aral Sea regions to identify patterns of evolution and circulation of the medieval biovar in this region in the 20th– 21st centuries. Based on the analysis of a complex of phylogenetic, epidemiological, epizootiological, climatic data, we reconstructed the paths of spread of 2.MED1 branch in the Northern Caspian region and in the Northern subzone of the Central Asian deserts.

## Materials and methods

### *Yersinia pestis* strains

This study examined *Y*. *pestis* strains that were isolated in the natural foci of the Northern Caspian and Northern Aral Sea regions from carriers and vectors of plague, as well as from humans (S1 Table). *Y*. *pestis* strains were received from the State Collection of Pathogenic Bacteria functioning at the premises of the Russian Research Anti-Plague Institute “Microbe” (Saratov, Russia). The strains were grown in liquid and agar LB medium (pH 7.2) for 24–48 hours at 28°C. The study of their cultural and morphological properties was performed in compliance with standard methods of laboratory diagnostics [9]. To determine the ability to ferment glycerol, 5 ml of Giss medium (1% peptone water, 1% Andrade indicator, pH7, 2) with 1% glycerol was seeded with 10^8^ CFU *Y*. *pestis* and cultured for 48 hours at a temperature of 28°C. The emergence of a red color of the medium indicated the ability of the studied strain to ferment glycerol. The ability to ferment the disaccharides rhamnose and arabinose was identified similarly, but instead of glycerol, 1% rhamnose or arabinose was added. To determine the denitrifying activity, 5 ml of LB broth with 0.1% potassium nitrate (KNO_3_) was seeded with 10^8^ CFU *Y*. *pestis* and incubated for 72 hours at 28°C. After adding the Griss reagent in strains with denitrifying activity, the medium acquired a raspberry color.

### Whole genome sequencing, SNPs identification, dendrogram construction

DNA of *Y*. *pestis* strains was isolated using PureLink Genomic DNA Mini Kit (Invitrogen, USA). Whole genome sequencing of *Y*. *pestis* strains was carried out using the Ion S5 XL System (Thermo Fischer Scientific) according to the manufacturer’s guide. Ion Xpress™ Plus Fragment Library Kit and Ion Xpress™ Barcode Adapter 1–16 Kit were taken for the primary preparation of samples. Ion Chef System, as well as Ion 520™ & Ion 530™ Kit–Chef and Ion 530™ Chip Kit were used for automated template preparation. The data processing and raw short-read sequences assembling *de novo* were accomplished using Ion Torrent Suit software package 5.10. and Newbler gsAssembler 2.6. The sequence reads were assembled into contigs with average coverage per genome being 98.47% (45,9- fold depth) and an average genome assembly size of 4,55 Mb (S2 Table). Core SNPs were identified by aligning contigs of *Y*. *pestis* strains to CO92 genome through Snippy 4.6. software program, then, 28 homoplastic SNPs were excluded (S3 Table). The resulting set of SNPs included only the core region of the genome. Using the Snippy software package (https://github.com/tseemann/snippy) provides a set of core SNPs that can be used for constructing high-resolution phylogenetic trees, since it excludes sites of possible recombination. The Maximum likelihood tree was constructed using software: Mesquite 3.6, PhyML-3.1, GTR model and 500 bootstrap replications. The Maximum parsimony tree was constructed in MEGA X (with 500 bootstrap replications).

## Results

For this work, 38 strains of *Y*. *pestis* from the foci of the Northern Caspian and Northern Aral Sea regions isolated over a period of more than a hundred years in 1912–2015 were studied. These strains are from the Caspian North-Western steppe (4 strains), Volga-Ural steppe (4), Volga-Ural sandy (9), Ural-Wil steppe (2), Ural-Emben desert (3), Pre-Ustyurt desert (1), North-Aral desert (4), Aral-Karakum desert (3), Caspian sandy (4), Dagestan plain-piedmont (2), as well as the Central-Caucasian high-mountain (1) and Zangezur-Karabakh high-mountain (1) foci. Strains were isolated from carriers: souslik *Spermophilus* (8 strains), midday gerbil *M*. *meridianus* (4), large gerbil *R*. *opinus* (5), vole *Microtus arvalis* (1); camel (1), fleas: *C*. *tesquorum*, *Radinopsylla cedetis*, *Ceratophyllus laeviceps*, *N*. *laeviceps* (7), ticks *Haemophisalus* (1), and from humans (11). Biochemical properties were analyzed for all strains, and whole genome sequencing was performed for 25 strains. The other 13 strains were sequenced by us earlier (S1 Table). All strains had typical *Y*. *pestis* cultural and morphological properties and were uniform in their biochemical features. They did not ferment rhamnose and melibiose, utilized glycerol, but did not reduce nitrates, which indicated that they belong to the medieval biovar.

**Fig 1:**
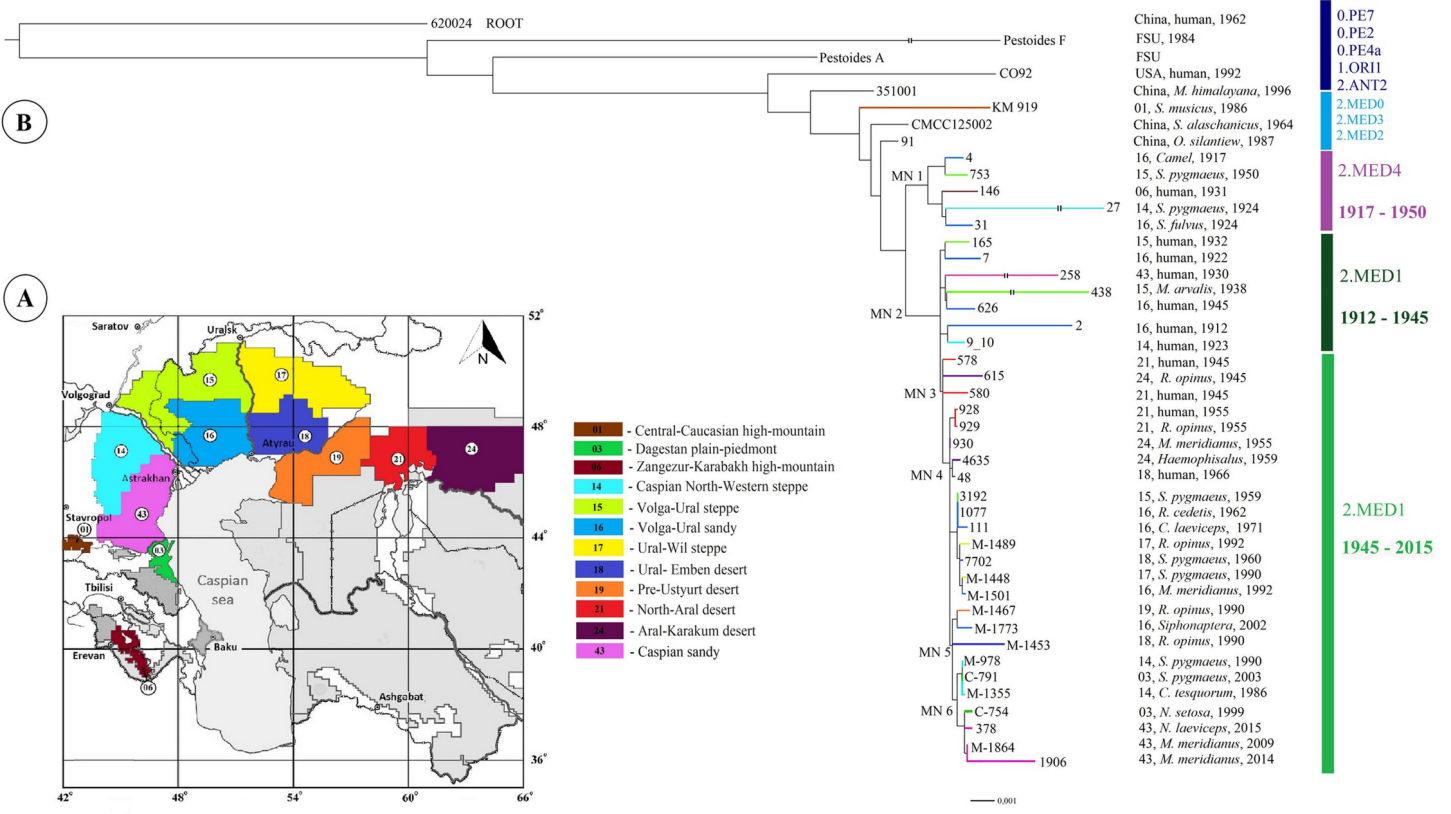
Phylogenetic analysis and population structure of *Y*. *pestis* strains of the medieval biovar (2.MED lineage) from natural foci of the Northern Caspian and Northern Aral Sea regions, other foci of the world. A. Map of natural plague foci of the Northern Caspian and Northern Aral Sea regions in Russia and Kazakhstan. The index number corresponds to the classification of foci, applied in Russia and other countries of the Commonwealth of Independent States: 1 –Central-Caucasian high-mountain, 3 –Dagestan plain-piedmont, 6 –Zangezur-Karabakh high-mountain, 14 –Caspian North-Western steppe, 15 –Volga-Ural steppe, 16 –Volga-Ural sandy, 17 –Ural-Wil steppe, 18 –Ural-Emben desert, 19 –Pre-Ustyurt desert, 21 –North-Aral desert, 24 –Aral-Karakum desert, 43 –Caspian sandy foci. B. Maximum Likelihood tree based on 1668 single nucleotide polymorphisms (SNPs) identified among 38 *Y*. *pestis* strains from the Northern Caspian and Northern Aral Sea regions, as well as 7 strains from other regions of the world. Branches are colored to indicate the focus of origin. SNPs in MN1-MN6 nodes are listed in the S4 Table.

To build a phylogenetic tree, the nucleotide sequences of all *Y*. *pestis* strains, taken in the study, were used. Nucleotide sequences of strains from other regions of the world, deposited in NCBI GenBank, were also deployed (S1 Table). According to the whole genome SNP analysis based on 1668 SNPs, dendrograms of phylogenetic relationships of the strains from the foci of the Northern Caspian and the Northern Aral Sea regions were constructed (Figs 1 and S2).

Strains from these foci were divided into three separate phylogenetic groups according to the time and place of their isolation (Fig 1). The first group consisted of strains that formed a separate phylogenetic branch, different from the 2.MED1 branch. We designated this branch as 2.MED4. It was not previously represented on the *Y*. *pestis* phylogenetic tree. This branch contains 9 unique SNPs located mainly in encoding sequences (Fig 1, MN1 –Medieval Node 1, S4 Table). 2.MED4 branch diverged from the trunk of the medieval biovar earlier than 2.MED1 branch and precedes it. In the dendrogram, 2.MED4 branch contains two clusters, which include four strains from the foci of the Northern and North-Western Caspian Sea regions (Volga-Ural sandy, Volga-Ural steppe, Caspian North-Western steppe foci) and one strain from the Zangezur-Karabakh autonomous focus within the Trans-Caucasian high-mountain focus. The strains were isolated in 1917–1950 at a considerable distance from each other, which indicates their widespread distribution in the first half of the 20th century in the Northern Caspian Sea region and possibly in Caucasus. Of the five 2.MED4 strains, one is isolated from a human corpse, the others are mainly from sousliks. This proves that 2.MED4 circulated in plague foci, and was also capable of causing death in humans. But no more 2.MED4 branch members were found among the available strains isolated in the second half of the 20th century.

The branch 2.MED4 is followed by the branch 2.MED1 on the dendrogram, which includes strains dated 1912–2015. It has 14 unique SNPs located in encoding sequences and in the intergenic space (Fig 1, MN2 node, S4 Table). Strains of the 2.MED1 branch are divided into two separate phylogenetic groups according to the spatial-temporal principle. The first of them consists of strains isolated in the foci of the Northern and North-Western Caspian Sea regions in the first half of the 20th century. This group is represented by three clusters, one of which includes strain (2) dated 1912 (human, Volga-Ural sandy focus) and strain (9) dated 1923 (human, Сaspian North-Western steppe focus). The second cluster consists of strains of 1922 and 1932, and the third–of 1930, 1938, 1945, from the Volga-Ural steppe, Volga-Ural sandy and Caspian sandy foci. Of seven strains in this group, six were isolated from humans during plague outbreaks. These strains were the etiological agents of plague outbreaks that occurred in the Northern and North-Western Caspian Sea regions in the early 20th century. We don’t exclude that these strains might have caused plague outbreaks that took place here in the late 19th century. The possibility of this assumption is confirmed by the continuous flow and similar nature of the plague outbreaks that took place here in the late 19th century till 1940th. In the middle of the 20th century, an inter-epizootic period occurred in the foci of the Northern Caspian Sea region, during which there were no cases of human infection and no epizootic activity was recorded in rodents. Epizootics began to reoccur only in the beginning of the second half of the 20th century. Strains of the second half of the 20th and early 21st centuries are included in the second phylogenetic group of 2.MED1 branch on the dendrogram. At the base of this group lies a sub-branch that differs from the sub-branch of the first half of the 20th century in 1 SNP in a gene encoding a hypothetical protein (Fig 1, MN3 node, S4 Table). This sub-branch is represented by strains of 1945 from the Northern Aral Sea region. Two of them were isolated from humans in the North-Aral desert focus in 1945. The first strain 578 was isolated in the Aral region of Kazakhstan on the island of Kug-Aral in October 1945 from a person with bubonic plague, and the second– 580 in the same area but on another island–Bigorundi in November 1945 from a person with primary pneumonic plague. Another strain 615 was isolated from a large gerbil in December 1945 in the Aral-Karakum desert focus in the village of Ak-Basti. All three strains are located on the sub-branch separately from each other. They differ in several SNPs specific to each strain: 578 –in 5 SNPs (4 SNPs in encoding sequences), 580 –in 8 SNPs in encoding sequences, 615– in 15 SNPs (13 SNPs in encoding sequences) (S4 Table). The sub-branch of strains from the Northern Aral Sea region of 1945 predates the 2.MED1 strains isolated later in the Northern Aral Sea region itself, as well as in the Northern Caspian region and Pre-Caucasus in the second half of the 20th and early 21st centuries.

From these Northern-Aral Sea strains of 1945 a sub-branch of the strains isolated in 1955–1991 extends. This new sub-branch differs from the sub-branch from the Northern Aral Sea region of 1945 in 3 SNPs, which are located in encoding sequences (Fig 1, MN4 node, S4 Table).

It is represented by a polytomy composed of both single strains and clusters of strains, combined according to the time and place of their isolation. The first on polytomy is a cluster of two strains from the North-Aral focus of 1955. Below, two strains that were isolated in the Aral-Karakum region in 1955 and 1959 depart from polytomy. These four strains are descendants of strains isolated in 1945 in the same region. Their phylogeny indicates that a stable population existed in the Northern Aral Sea region at least from 1945 to 1959, which caused outbreaks and cases of plague. At the beginning of the second half of the 20th century strains from the Northern Aral Sea region entered the Northern Caspian region forming persistent plague foci along the way of their spread from East to West. The monophyletic clade of the entire sub-branch indicates that colonization of the Northern Caspian Sea region in the second half of the 20th century with the strains from the foci of the Northern Aral Sea region was a one-time event.

Further on, the polytomy contains a separate cluster of strains from four foci of the Northern Caspian Sea region: Ural–Wil steppe, Ural-Emben desert, Volga-Ural sandy, Volga-Ural steppe, isolated from carriers–small susliks, gerbils and their fleas. The cluster comprises both, earlier single strains (1959, 1963, and 1971) and a sub-cluster of later strains from 1962 to 1991. This means that in the second half of the 20th century, a separate population of closely related strains, whose ancestors were the strains from the Northern Aral Sea region, dated back 1945, took root and existed in the territory of four neighboring foci of the Northern Caspian Sea region in 1959–1991.

At the end of the 20th century this polytomy, which included strains from the Northern Aral Sea (1955–1959) and the Northern Caspian Sea (1959–1991) regions, gave rise to a new sub-branch (Fig 1, MN5 node, S4 Table), differing in 1 SNP in the intergenic space. This new sub-branch includes 1990–2002 strains from the North-Eastern (Pre-Ustyurt desert focus) and Northern (Volga-Ural sandy, Ural-Emben desert foci) Caspian Sea regions. This sub-branch, in its turn, precedes the strains isolated in the late 20th and early 21st centuries in the North-Western Caspian and Pre-Caucasus (Fig 1, MN6 node, S4 Table), 2 SNPs in encoding sequences. The strains combine two clusters, including a cluster of strains from the Caspian North-Western steppe and Dagestan plain-piedmont (Pre-Caucasus) foci dated 1986–1999. Another cluster is predominantly represented by the strains of the 21^st^ century (1999–2015) from the Caspian sandy and Dagestan plain-piedmont foci.

**Fig 2:**
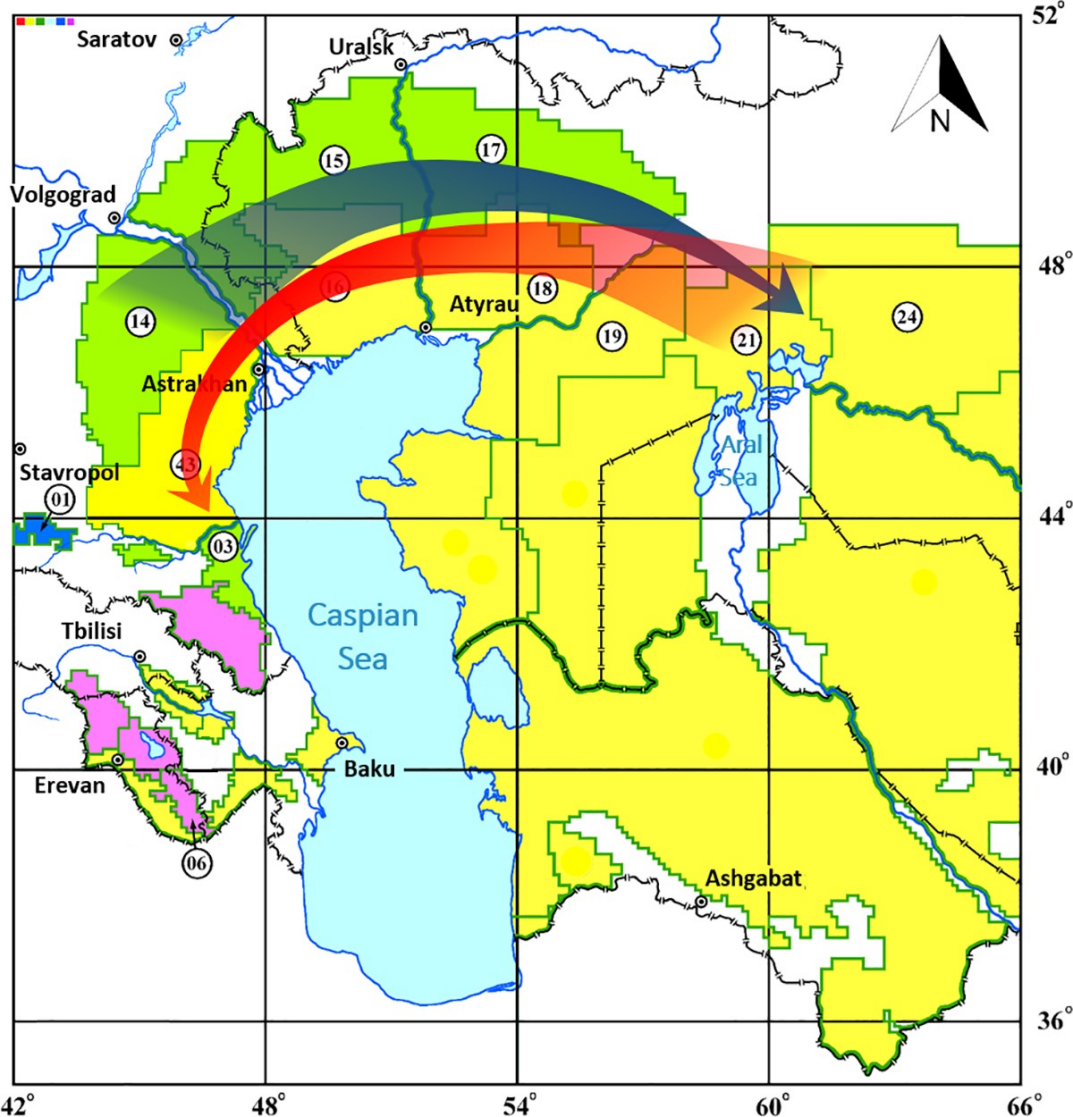
Directions of spread of *Y*. *pestis* of medieval biovar, phylogenetic branch 2.MED1 in the Northern Caspian and Northern Aral Sea regions in the 20th-21st centuries. The blue arrow indicates the direction of spread of the 2.MED1 branch in the first half of the 20th century, the red arrow–in the second half of the 20th-early 21st century. Green color marks plain and low-mountain foci of the souslik type, yellow–plain and low-mountain foci of the gerbil type, blue–high-mountain foci of the souslik type, pink–high-mountain foci of the vole type. The index number corresponds to the classification of foci, applied in Russia and other countries of the Commonwealth of Independent States: 1 –Central-Caucasian high-mountain, 3 –Dagestan plain-piedmont, 6 –Zangezur-Karabakh high-mountain, 14 –Caspian North-Western steppe, 15 –Volga-Ural steppe, 16 –Volga-Ural sandy, 17 –Ural-Wil steppe, 18 –Ural-Emben desert, 19 –Pre-Ustyurt desert, 21 –North-Aral desert, 24– Aral-Karakum desert, 43 – Caspian sandy foci.

Thus, the constructed dendrogram sequentially and strain-by-strain reflects the direction of spread of the medieval biovar in the Northern Caspian and Northern Aral Sea regions in the 20th-21st centuries, as well as accompanying this spread sequential evolution of the 2.MED1 branch. In the dendrogram, branch 2. MED1 is preceded by branch 2.MED4. In the first half of the 20th century, both populations circulated in the foci of the Northern Caspian Sea region. 2.MED1 population was the etiological agent of outbreaks occurring in that region. After a long inter-epizootic period at the beginning of the second half of the 20th century, strains related to the strains of the first half of the 20th century began to be isolated in the territories, but their nearest predecessors were strains from the Northern Aral Sea region dated 1945. From this North-Aral Sea population, the modern strains of 2.MED1 evolved, which in the second half of the 20th century reached the North-Eastern and Northern Caspian Sea regions, then spread to the North-Western Caspian Sea region and Pre-Caucasus. This led to the activation of plague foci of the Northern, North-Western Caspian and Pre-Caucasus regions after a long inter-epizootic period. A hypothetical scheme of circulation of *Y*. *pestis* 2.MED1 strains of medieval biovar in the Northern Caspian and Northern Aral Sea regions in the 20th-21st centuries is shown in Fig 2.

## Discussion

Strains of the medieval biovar, phylogenetic lineage 2.MED belong to highly virulent and epidemically significant strains of *Y*. *pestis*. Previously, it was thought that the medieval biovar was the etiological agent of the second plague pandemic, which began in medieval Europe (The Black Dearth, 1346–1353 AD), claimed millions of human lives, and lasted until the 18th century. However, the data from reconstruction of archaeogenomes from the second pandemic period did not confirm this hypothesis and showed that this pandemic was caused by the strains belonging to the phylogenetic lineage that was a precursor to strains associated with the third plague pandemic [10–13]. The medieval biovar seems to have spread afterwards in the 19-20^th^ centuries. At the beginning of the 20th century, it caused numerous outbreaks in the foci of the Caspian Sea region in Russia, Kazakhstan, and Turkmenistan, and then spread to the territory of other Central Asian Republics with the formation of sustained foci. There is no earlier information about the manifestations of plague in the desert landscapes of Central Asia. Recently, it was shown that the 2.MED lineage had the highest spread velocity compared to other *Y*. *pestis* lineages, including 1.ORI strains of the oriental biovar that caused the third plague pandemic [14]. It should be noted that the medieval biovar has high adaptive properties and exists in different types of foci–steppe, desert, mountain, high-mountain, including territories with highly arid climate. The reasons for such a high adaptability of the medieval biovar, apparently, are associated with the changes in the genome, which allow it to survive in various landscapes.

The 2.MED branch of the medieval biovar is one of the most recently divergent and monomorphic branches of *Y*. *pestis*, which genetic typing is a difficult task. Available online resources, such as whole genome sequence typing database BacWGSTdb 2.0, can be very useful for source tracking of clinical cases and analyzing epidemic outbreaks of *Y*. *pestis* in a globalized community [15]. It provides a one-step solution for rapid classification and source tracking, as well as for searching closely related strains deposited in a publicly available database.

This study provides phylogenetic analysis of the data from whole genome sequencing of *Y*. *pestis* strains of the medieval biovar, isolated over a period of more than a hundred years (1912–2015) in the foci of the Northern Caspian and Northern Aral Sea regions (Russia, Kazakhstan). The sequential evolution of the medieval biovar in these territories has been established. The branch 2.MED4 of *Y*. *pestis* was identified. The 2.MED4 population existed in the foci of the Northern and North-Western Caspian Sea regions in the first half of the 20th century and could cause plague in humans. 2.MED1 strains became the etiological agent of plague outbreaks in the Caspian Sea region since at least the beginning of the 20th century. The maximum incidence was registered in the 1930s and early 1940s. The results of phylogenetic analysis show that 2.MED1 population that existed in the early 20th century in the North-Western Caspian region had greatly expanded its area eastwards in the Caspian lowland by the 1940s. This led to the rooting of 2.MED1 in 1940s-1950s in the Northern Aral Sea region. Area expansion of the population in the Caspian lowland in the first half of the previous century occurred against the background of increased humidification of the Northern Caspian region and preservation of high level of the Caspian Sea (S1 Appendix) [16, 17]. The spread of 2.MED1 in the Northern Aral region in the 1940-1950s also occurred against the background of the high level of the Aral Sea in the first half of the 20th century, which had a positive impact on the ecosystems of the Northern subzone of deserts of the Eastern Caspian Sea region [18, 19]. But towards the middle of the 20th century, epidemiological and epizootiological manifestations in the Northern Caspian Sea region stopped. We assume that one of the main causes of this was climate warming which began in the 1930s-1940s and covered vast areas of steppe and desert landscapes of the Caspian Sea region. The Caspian Sea is the world’s largest inland water body, with an area of about 370,000 square kilometers. During periods of global warming, the level of the Caspian Sea decreases due to the intense evaporation of water from its surface. In 1930–1970, the level of the Caspian Sea fell sharply by more than three meters, which had a dramatic impact on the ecosystem of the Caspian region [16, 17]. Climate aridization in the Caspian Sea region in the 1940s-1950s resulted in the ending of plague epizootics in the regions of the Northern, North-Western Caspian Sea and Pre-Caucasus for decades [20]. The impact of climate factors was significantly enhanced by the unprecedented measures taken to control plague foci in this region. In contrast, at the same time, active epizootics and outbreaks of plague occurred in the Northern Aral Sea region.

Hereinafter, activation of the foci in the Northern, North-Western Caspian and Pre-Caucasus regions which occurred in 1960s-1970s after a long inter-epizootic period in the middle of the 20th century was caused by the successive spread of 2.MED1 strains from the foci of the Northern Aral Sea region (North-Aral and Aral-Karakum desert foci, 1945) through the foci of the North-Eastern and Northern Caspian regions toward the North-Western Caspian region and Pre-Caucasus. We suppose that this reverse movement of 2.MED1 was caused by another cyclical change in climatic conditions in the Northern Caspian and Northern Aral Sea regions. During the next two decades after 1977, there was an unexpected rapid increase in the level of the Caspian Sea by 2.5 m, reaching a maximum in 1995 [16, 17]. The decrease in aridity of the climate and the rise of the Caspian Sea level again created conditions for the emergence of 2.MED1 foci in the Northern Caspian region after decades of absence of plague, and for further 2.MED1 distribution in the Pre-Caucasus.

The dendrogram of phylogenetic relationships of 38 *Y*. *pestis* strains presented in this study reflects the sequential evolution of 2.MED lineage on a temporal-geographical basis and allows us to carry out a hypothetical reconstruction of the circulation of 2.MED1 branch in the foci of the Northern Caspian and the Northern Aral Sea region in the 20th-21st centuries (Fig 2). It should be noted that the evolution of strains in the Northern Caspian and Northern Aral Sea regions in the second half of the 20th and early 21st centuries was not accompanied by a significant change in the genome, but only by the emergence of a small number of SNPs (1–3) mainly in coding sequences. Apparently, this is due to the strict control of changes in the genome under the conditions of plague foci. In the second half of the 20th—early 21st century, there were no outbreaks of plague in the Northern Caspian Sea region. Only single cases of human plague associated with field work on epizootic territories were recorded.

More dramatic evolutionary events occurred in the Northern Caspian Sea region at the beginning of the 20th century, when many strains of 2.MED4 and 2.MED1populations had a large number of SNPs (Fig 1, S3 Table). This could result from adaptation to new conditions (epidemic outbreaks, new territories), or other not yet established factors. In General, the results obtained in this study indicate the prospects of complex use of phylogenetic, epidemiological and epizootiological data in combination with climate data to identify spatial and temporal patterns of evolution and circulation of the medieval biovar of *Y*. *pestis* in the foci of Eastern Europe and Central Asia in the XX-XXI centuries.

## Supporting information

S1 Table Strains of *Yersinia pestis* used in this study. (XLSX)

S2 Table Data production. (XLSX)

S3 Table Genomes used for core SNP calling and phylogenetic analysis of *Y*. *pestis* of 2.MED lineage. (XLSX)

S4 Table SNPs, marker for the key nodes of the maximum likelihood tree of *Y*. *pestis* strains, used in this study. (XLSX)

S1 Fig Map of natural plague foci of the Northern Caspian and Northern Aral Sea regions in Russia and Kazakhstan. Green color marks plain and low-mountain foci of the suslik type, yellow–plain and low-mountain foci of the gerbil type, blue–high-mountain foci of the suslik type, pink–high-mountain foci of the vole type. The index number corresponds to the classification of foci, applied in Russia and other countries of the Commonwealth of Independent States: 1 –Central-Caucasian high-mountain, 3– Dagestan plain-piedmont, 6 –Zangezur-Karabakh high-mountain, 14 –Caspian North-Western steppe, 15 –Volga-Ural steppe, 16 –Volga-Ural sandy, 17 –Ural-Wil steppe, 18 –Ural-Emben desert, 19 –Pre-Ustyurt desert, 21 –North-Aral desert, 24– Aral-Karakum desert, 43 – Сaspian sandy.
(TIF)

S2 Fig Phylogenetic analysis of *Y*. *pestis* strains of the medieval biovar (2.MED lineage) from natural foci of the Northern Caspian and Northern Aral Sea regions, other foci of the world. Maximum Parsimony tree based on 1668 single nucleotide polymorphisms (SNPs) identified among 38 *Y*. *pestis* strains from the Northern Caspian and Northern Aral Sea regions, as well as 7 strains from other regions of the world. Constructed in MEGA X.
(TIF)

S1 Appendix The Caspian Sea and Aral Sea level fluctuation in the 20th-21st centuries. (DOCX)

## References

[1] Natural plague foci in the territory of Caucasus, Caspian-Sea Region, Central Asia and Siberia. Edited by Onishchenko GG, Kutyrev VV. Moscow: “Medicine”. 2004.

[2] Cadastre of epidemic and epizootic manifestations of plague in the territory of the Russian Federation and neighboring countries (1876–2015). Edited by Kutyrev VV, Popova AYu. Saratov: “Amirit”. 2016.

[3] Eroshenko GA, Popov NV, Al’khova ZhV, Balykova AN, Kukleva LM, Kutyrev VV. Phylogenetic analysis of *Yersinia pestis* strains of medieval biovar, isolated in the Precaspian North-Western steppe plague focus in the XX century. Problems of Particularly Dangerous Infections. 2019; 2:55–61. 10.21055/0370-1069-2019-2-55-61

[4] Achtman M, Zurth K, Morelli G, Torrea G, Guiyoule A, Carniel E. *Yersinia pestis*, the cause of plague, is a recently emerged clone of *Yersinia pseudotuberculosis*. Proc Natl Acad Sci U S A. 1999; 96:14043–14048. 10.1073/pnas.96.24.14043 PMCID: PMC24187. 10570195 PMC24187

[5] Morelli G, Song Y, Mazzoni CJ, Eppinger M, Roumagnac P, Wagner DM, et al *Yersinia pestis* genome sequencing identifies patterns of global phylogenetic diversity. Nat Genet. 2010; 42(12):1140–1143. Epub 2010/11/03. [pii] 10.1038/ng.705 . 21037571 PMC2999892

[6] Cui Y, Yu C, Yan Y, Li D, Li Y, Jombart T, et al Historical variations in mutation rate in an epidemic pathogen, *Yersinia pestis*. Proc Natl Acad Sci U S A. 2013; 110(2):577–582. Epub 2012/12/29. 10.1073/pnas.1205750110 23271803 PMC3545753

[7] Kutyrev VV, Eroshenko GA, Motin VL, Nosov NY, Krasnov JM, Kukleva LM, et al Phylogeny and classification of *Yersinia pestis* through the lens of strains from the plague foci of Commonwealth of Independent States. Front Microbiol. 2018; 9:1106 Epub. 2018/5/25. 10.3389/fmicb.2018.01106 29887859 PMC5980970

[8] Kukleva LM, Shavina NYu, Odinokov GN, Oglodin EG, Nosov NYu, Vinogradova NA et al Analysis of diversity and identification of the genovariants of plague agent strains from Mongolian foci. Rus J Genet. 2015; 51:238–244. 10.1134/S1022795415010068. 26027368

[9] Laboratory diagnostics of dangerous infectious diseases. Practice guidelines. Edited by Onishchenko GG, Kutyrev VV. Moscow: “Shiko” 2013.

[10] Haensch S, Bianucci R, Signoli M, Rajerison M, Schultz M, Kacki S, et al Distinct clones of *Yersinia pestis* caused the Black Death. PLoS Pathog. 2010 10; 6(10): e1001134 10.1371/journal.ppat.1001134 PMCID: PMC2951374. 20949072 PMC2951374

[11] Bos KI, Schuenemann VJ, Golding GB, Burbano HA, Waglechner N, Coombes BK, et al A draft genome of *Yersinia pestis* from victims of the Black Death. Nature. 2011 10 12; 478(7370):506–10. 10.1038/nature10549 21993626 PMC3690193

[12] Seifert L, Wiechmann I, Harbeck M, Thomas A, Grupe G, Projahn M, et al Genotyping *Yersinia pestis* in historical plague: evidence for long-term persistence of *Y*. *pestis* in Europe from the 14th to the 17th century. PLoS One. 2016 1 13; 11(1):e0145194 10.1371/journal.pone.0145194 eCollection 2016. 26760973 PMC4712009

[13] Spyrou MA, Keller M, Tukhbatova RI, Scheib CL, Nelson EA, Andrades Valtueña A, Phylogeography of the second plague pandemic revealed through analysis of historical *Yersinia pestis*genomes. Nat Commun. 2019 10 2; 10(1):4470 10.1038/s41467-019-12154-0 31578321 PMC6775055

[14] Xu L, Stige LC, Leirs H, Neerinckx S, Gage KL, Yang R et al Historical and genomic data reveal the influencing factors on global transmission velocity of plague during the Third Pandemic. Proc Natl Acad Sci U S A. 2019; 116(24):11833–11838. 10.1073/pnas.1901366116 31138696 PMC6584904

[15] Ruan Z, Feng Y. BacWGSTdb, a database for genotyping and source tracking bacterial pathogens. Nucleic Acids Res. 2016 1 4; 44(D1):D682–687. 10.1093/nar/gkv1004 Epub 2015 Oct 3. 26433226 PMC4702769

[16] Kasimov NS, Gennadiev AN, Kasatenkova MS, Lychagin MY, Kroonenberg SB, Koltermann P. Geochemical changes in the Caspian salt marshes due to the sea level fluctuations. Earth Science Research. 2012; 1(2):262–278. 10.5539/esr.v1n2p262

[17] Chen J, Tapley BD, Wilson SR, Kostianoy AG. Long-term Caspian sea level change. Geophysical Research Letters. 2017; 6 10.1002/2017GL073958

[18] Bortnik VN. (1996) Changes in the water-level and hydrological balance of the Aral Sea InMicklin P.P., Williams W.D. (eds) The Aral Sea Basin. NATO ASI Series, (Series 2. Environment), Vol. 12 Springer, Berlin, Heidelberg pp. 25–32 10.1007/978-3-642-61182-7_3

[19] Cretaux J-F, Letolle R, Bergé-Nguyen M. History of Aral Sea level variability and current scientific debates. Global and Planetary Change. 2013; 110: 99–113. 10.1016/j.gloplacha.2013.05.006

[20] Popov NV, Udovikov AI, Eroshenko GA, Karavaeva TB, Yakovlev SA, Porshakov AM, et al Impact of the Caspian Sea level fluctuations on the epizootic activity of the Caspian sandy natural plague focus. Med Parazitol (Mosk). 2016; Jan-Mar; 1:12–17. . 27029140

