# Supplementary figures and images for: Evolution and circulation of Yersinia pestis in the Northern Caspian and Northern Aral Sea regions in the 20th-21st centuries
Source: PLoS One. 2021 Feb 11;16(2):e0244615. doi: 10.1371/journal.pone.0244615 (PMC7878065; doi:10.1371/journal.pone.0244615)

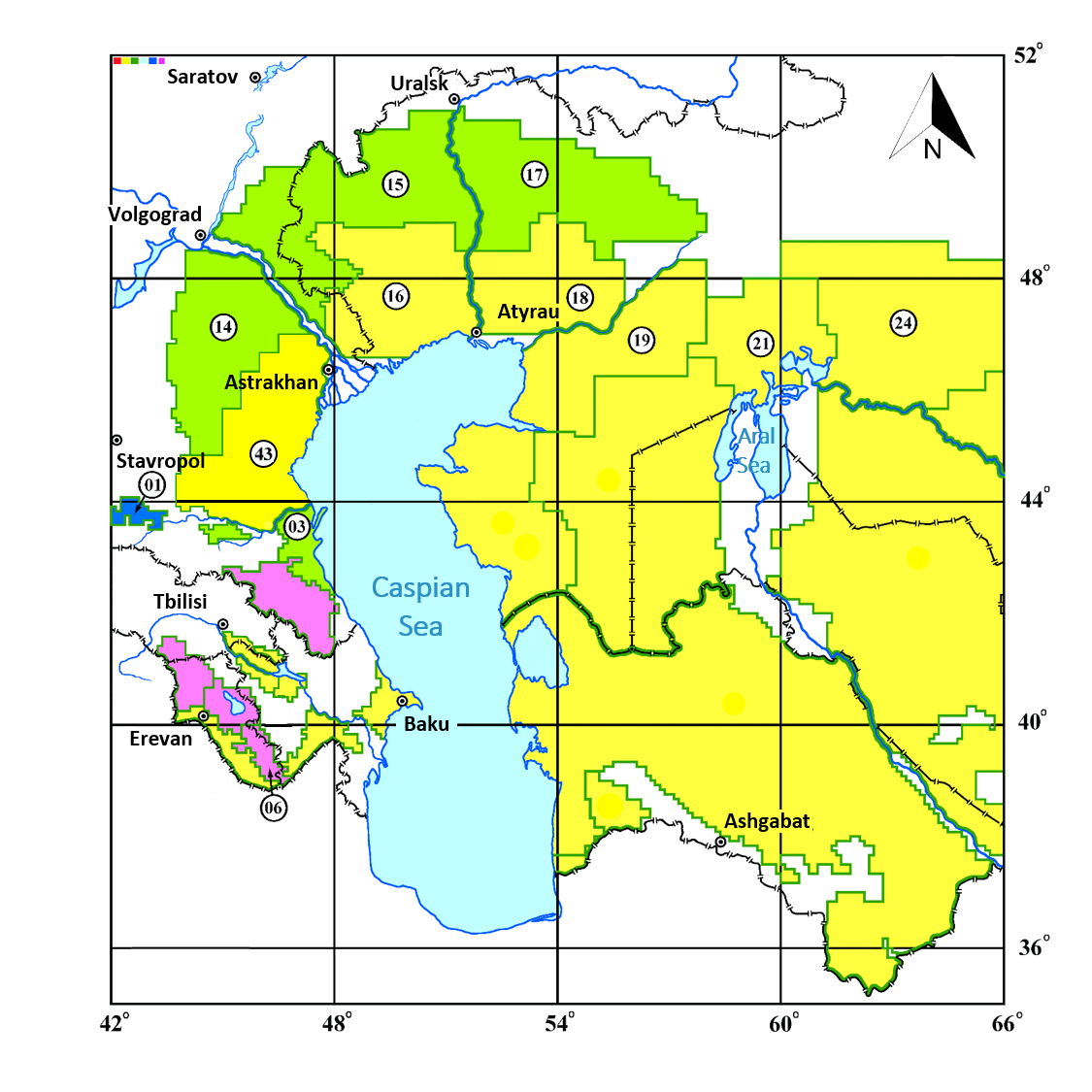

Supplement: S1 Fig — Green color marks plain and low-mountain foci of the suslik type, yellow–plain and low-mountain foci of the gerbil type, blue–high-mountain foci of the suslik type, pink–high-mountain foci of the vole type. The index number corresponds to the classification of foci, applied in Russia and other countries of the Commonwealth of Independent States: 1 –Central-Caucasian high-mountain, 3– Dagestan plain-piedmont, 6 –Zangezur-Karabakh high-mountain, 14 –Caspian North-Western steppe, 15 –Volga-Ural steppe, 16 –Volga-Ural sandy, 17 –Ural-Wil steppe, 18 –Ural-Emben desert, 19 –Pre-Ustyurt desert, 21 –North-Aral desert, 24– Aral-Karakum desert, 43 – Сaspian sandy. (TIF) [file pone.0244615.s005.tif]

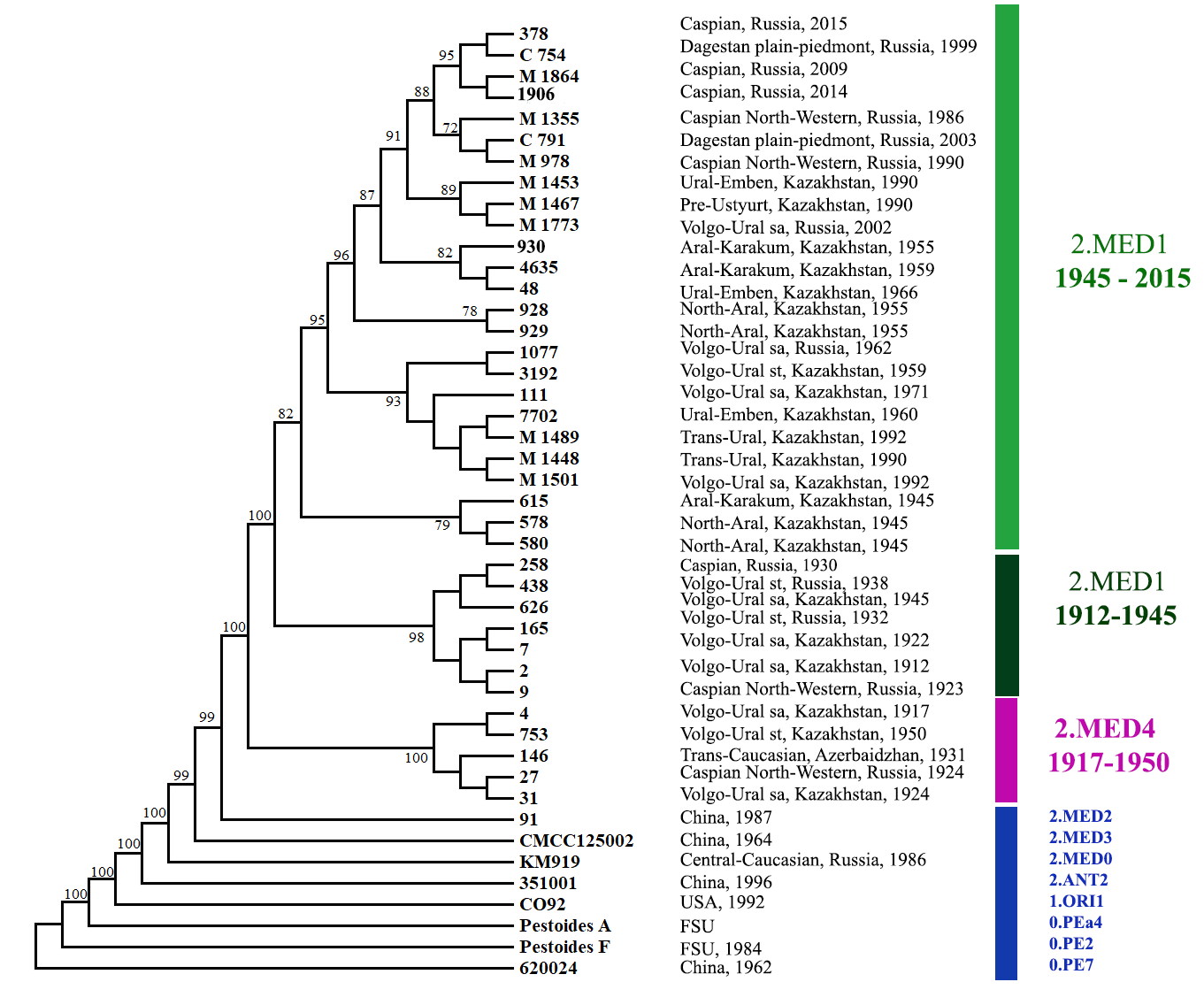

Supplement: S2 Fig — Maximum Parsimony tree based on 1668 single nucleotide polymorphisms (SNPs) identified among 38 Y. pestis strains from the Northern Caspian and Northern Aral Sea regions, as well as 7 strains from other regions of the world. Constructed in MEGA X. (TIF) [file pone.0244615.s006.tif]
