# Supplementary material for: Evolution and circulation of Yersinia pestis in the Northern Caspian and Northern Aral Sea regions in the 20th-21st centuries
Source: PLoS One. 2021 Feb 11;16(2):e0244615. doi: 10.1371/journal.pone.0244615 (PMC7878065; doi:10.1371/journal.pone.0244615)
Supplement: S1 Appendix — (DOCX) [file pone.0244615.s007.docx]

S1 Appendix

The Caspian Sea and Aral Sea level fluctuation in the 20^th^-21st centuries





Figure 1. The Caspian Sea level fluctuations in the XX century [Kasimov NS, Gennadiev AN, Kasatenkova MS, Lychagin MY, Kroonenberg SB, Koltermann P. Geochemical Changes in the Caspian Salt Marshes Due to the Sea Level Fluctuations. Earth Science Research. 2012; 1(2), 262-278. http://dx.doi.org/10.5539/esr.v1n2p262]


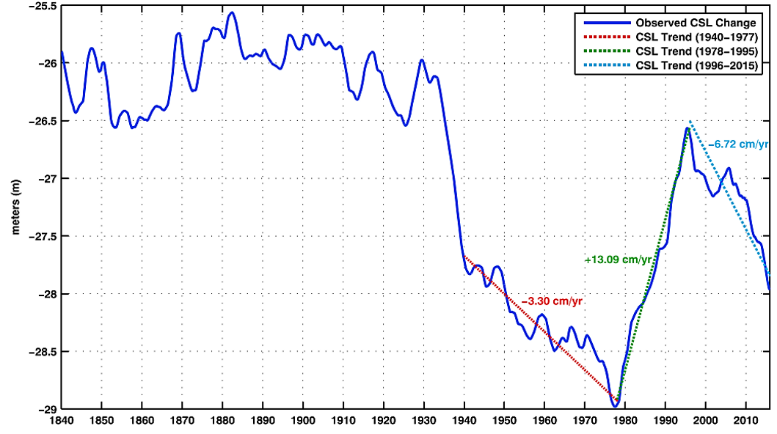


Figure 2. Monthly mean Caspian Sea level changes observed by tide gauges (1940–1997) and satellite altimetry (1997 to 2015, provided by Legos/CNEShttp://hydroweb.theia-land.fr/) [Chen J, Tapley BD, Wilson SR, Kostianoy AG. Long-Term Caspian Sea Level Change. Geophysical Research Letters. 2017; June. DOI: 10.1002/2017GL073958]





Figure 3. Water-level fluctuations in the Aral Sea. Dashed line, from reconstruction; solid line, from instrumental data [Bortnik VN. (1996) Changes in the water-level and hydrological balance of the Aral Sea. In Micklin P.P., Williams W.D. (eds) The Aral Sea Basin. NATO ASI Series, (Series 2. Envi-ronment), Vol. 12. Springer, Berlin, Heidelberg. pp. 25-32 https://doi.org/10.1007/978-3-642-61182-7_3].





Fig. 4. Water level variations of the Aral Sea, from 1950 to 2010, inferred from in situ measurements until 2000 and from satellite altimetry from 1992 to 2010 [Cretaux J-F, Letolle R, Bergé-Nguyen M. History of Aral Sea level variability and current scientific debates. Global and Planetary Change. 2013; 110: 99–113. http://doi.org/10.1016/j.gloplacha.2013.05.006]
